# The roles of KRAS in cancer metabolism, tumor microenvironment and clinical therapy

**DOI:** 10.1186/s12943-024-02218-1

**Published:** 2025-01-13

**Authors:** Qinglong Ma, Wenyang Zhang, Kongming Wu, Lei Shi

**Affiliations:** 1 RNA Oncology Group, School of Public Health, Lanzhou University, Lanzhou, 730000 People’s Republic of China; 2 Cancer Center, Shanxi Bethune Hospital, Shanxi Academy of Medical Science, Tongji Shanxi Hospital, Third Hospital of Shanxi Medical University, Taiyuan, 030032 People’s Republic of China; 3 Cancer Center, Tongji Hospital of Tongji Medical College, Huazhong University of Science and Technology, Wuhan, 430030 People’s Republic of China; 4 Cancer Research UK Manchester Institute, The University of Manchester, Wilmslow Road, Manchester, M20 4BX UK

**Keywords:** KRAS, Cancer metabolism, Tumor microenvironment, Epigenetic modification

## Abstract

KRAS is one of the most mutated genes, driving alternations in metabolic pathways that include enhanced nutrient uptaking, increased glycolysis, elevated glutaminolysis, and heightened synthesis of fatty acids and nucleotides. However, the beyond mechanisms of KRAS-modulated cancer metabolisms remain incompletely understood. In this review, we aim to summarize current knowledge on KRAS-related metabolic alterations in cancer cells and explore the prevalence and significance of KRAS mutation in shaping the tumor microenvironment and influencing epigenetic modification via various molecular activities. Given that cancer cells rely on these metabolic changes to sustain cell growth and survival, targeting these processes may represent a promising therapeutic strategy for KRAS-driven cancers.

## Introduction

Cancer is a complex and multifaceted disease characterized by the uncontrolled growth and spread of abnormal cells. It can develop in virtually any tissue or organ in the body and is driven by genetic mutations, environmental factors, and cellular disruptions [1, 2]. The progression of cancer involves a series of changes, including rapid cell growth, resistance to cell death, and the capacity to invade surrounding tissues and metastasize to distant sites. Tumor suppressors and oncogenes are two critical types of genes that play central roles in the regulation of cell growth and division [3, 4]. Tumor suppressor genes act as the "brakes" of the cells, preventing uncontrolled cell proliferation. These genes, including p53 and RB1, encode proteins that regulate cell cycle progression, promote DNA repair, and enhance apoptosis [5, 6]. Conversely, oncogenes are the "fuel pedals" of the cells, driving cell growth, division, and survival [7–9]. The RAS gene family, including KRAS, NRAS, and HRAS, plays a crucial role in cellular signaling pathways that facilitate growth, differentiation, and survival [10]. Among them, KRAS mutations are particularly common in several cancers such as pancreatic, colorectal, and lung cancers [11, 12].

Over the past two decades, tumor metabolism has emerged as a critical hallmark of cancer, significantly contributing to tumor development and progression [13–15]. Tumor metabolism refers to the unique reprogramming of metabolic processes in cancer cells to support their rapid growth, proliferation, and survival. Unlike normal cells, cancer cells undergo metabolic reprogramming to meet the increased demands for energy, macromolecule biosynthesis, and redox balance [16, 17]. One of the most well-known tumor metabolisms is Warburg effect, which tumor cells preferentially utilizing glycolysis for energy production, followed by lactate fermentation in the cytosol, rather than relying on the more efficient process of oxidative phosphorylation in the mitochondria, even in the presence of oxygen [18, 19]. Additionally, mutations in mitochondrial DNA or alterations in mitochondrial enzymes can further modify metabolic pathways to favor tumor growth [20, 21]. Cancer cells also frequently alter lipid metabolism by increasing de novo fatty acid synthesis, enhancing the uptake of exogenous lipids, and remodeling lipid to produce membranes, thereby supporting rapid proliferation [22, 23]. Moreover, cancer cells exhibit increased dependence on glutamine, an amino acid that provides essential critical carbon and nitrogen sources [24, 25]. Other amino acids, such as serine, glycine, and asparagine, are also crucial for tumor growth [26, 27]. In summary, tumor metabolism is a fundamental aspect of cancer biology, enabling cancer cells to sustain rapid growth and survive in the challenging tumor microenvironment. Understanding these specific metabolic alterations provides deep insights into tumor biology and highlights potential targets for therapeutic intervention.

KRAS mutations significantly rewire cellular metabolic processes to meet the increased energy demands of cancer cells. In this review, we delve into recent advancements to understand KRAS mutant in metabolic reprogramming, emphasizing the critical role of KRAS-driven metabolism in tumor microenvironment. These insights may pave the way for developing targeted therapies against KRAS-driven cancers.

## KRAS

Kirsten rat sarcoma viral oncogene homolog (KRAS) is a small GTPase that functions as a molecular switch within cells [28]. KRAS cycles between two conformational states: an inactive GDP-bound state and an active GTP-bound state. This cycling is regulated by GTPase-activating proteins (GAPs) and guanine nucleotide exchange factors (GEFs), respectively. In the GTP-bound active form, KRAS undergoes a conformational change that enables it to interact with and activate various downstream signaling pathways, such as MAPK/ERK pathway, PI3K/AKT pathway, and RalGDS pathway [11, 29].

KRAS is one of the most frequently mutated oncogenes in human cancers, particularly in pancreatic, colorectal, and lung cancers. These mutations typically occur in codons 12, 13, or 61, resulting in the active GTP-bound state by impairing the ability of KRAS to hydrolyze GTP to GDP [30]. Targeting KRAS has been a significant challenge in cancer therapy due to its structure, which lacks easily druggable pockets, making it difficult for conventional small molecules to bind effectively. However, recent advances have led to the development of inhibitors that specifically target KRAS G12C inhibitors (Sotorasib and Adagrasib) [31, 32]. Despite these breakthroughs, resistance to KRAS-targeted therapies remains a significant obstacle, and combination therapies may be a promising strategy to overcome this resistance.

## KRAS and cancer metabolism

Cancer metabolism refers to the distinct and often reprogrammed metabolic processes in cancer cells, which significantly differ from those in normal cells. These alterations are crucial for supporting the rapid growth, survival, and proliferation of cancer cells by meeting their increased demands for energy, biosynthetic precursors, and redox balance. Based on the fundamental roles of KRAS in cancer, understanding the correlation between KRAS and metabolic changes, such as the Warburg effect, mitochondrial metabolism, lipid metabolism, glutamine metabolism is vital for deciphering tumor biology and developing targeted therapies.

### KRAS and the Warburg effect

The Warburg effect, proposed by Otto Warburg, is a process where cancer cells preferentially produce energy through increased glycolysis, followed by lactic acid fermentation and lactate secretion. This process, also known as aerobic glycolysis, produces less ATP per glucose molecule compared to oxidative phosphorylation but operates much faster, enabling cancer cells to rapidly generate energy to support the accelerated growth and proliferation [18]. The role of the KRAS in metabolic reprogramming was initially uncovered by its ability to enhance glycolysis. This unique function allows tumor cells to more efficiently metabolize glucose, providing both energy and the molecular precursors for biomass synthesis (Table 1). Consequently, tumor cells gain the capacity for uncontrolled proliferation and growth [33].

**Table 1:** KRAS and glycolysis in cancer

Cancer Type	Targets	Function and Mechanism	Ref
Pan-cancer	HK1	KRAS4A interacts with HK1 on mitochondria to prevent the metabolic inhibition of HK1 and drive glucose uptake.	[34]
PDAC	COX-2	COX-2 ablation attenuates KRAS hyperactivation, leading to reversal of aerobic glycolysis and high allosteric proliferation in response to HFD attack.	[35]
PDAC	GFPT1	KRAS G12D enhances glycolytic flux, activates hexosamine biosynthesis and ribose biogenesis by promoting HK1, HK2, PFK1 and LDHA.	[36]
PDAC	GM3, SM4	KRAS G12V enhances glycolysis and glycosphingolipids biosynthesis, which in turn, stabilizing KRAS plasma membrane localization and nanoscale spatial organization.	[37]
PDAC	JAK1/STAT6/MYC	KRAS G12D upregulates Type I Cytokine Receptor IL2Rγ and IL4R, leading the activation of JAK1/STAT6/MYC and rewiring the glycolytic reprogramming.	[38]
PDAC	PRMT1	PRTM1 enhances the chromatin accessibility of HK2 and SLC2A1, in order to promote glycolysis in PDAC tumorigenesis.	[39]
PDAC	5-HT	5-HT promotes cell growth and glycolytic flux via MYC and HIF1A in KRAS-driven PDAC.	[40]
PDAC	Nutri-hijacker	Nutri-hijacker reshape the tumor microenvironment induces autophagy and suppresses PDAC growth via impairing glycolysis and restraining glutaminolysis.	[41]
PDAC	NOX4	p16-Rb-E2F-induced NOX4 contributes glycolysis via NAD^+^ in KRAS G12V-driven PDAC.	[42]
PDAC	CA9	KRAS G12D induces glycolysis and enhances tumor metastasis via CA9.	[43]
PDAC	NIX	NIX represses mitochondrial content, restrains mitophagy and glucose flux in KRAS G12D-driven PDAC,	[44]
PDAC	ATG7	Lacking p53 and ATG7 enhances glycolytic and pentose phosphate pathway in KRAS G12D-driven PDAC.	[45]
PAAD/NSCLC	LIFR	LIFR activates STAT3, inhibits glycolysis in KRAS-driven cancer cells.	[46]
NSCLC	FBP1	NK cells prevent KRAS-driven lung cancer initiation via suppression of FBP1.	[47]
NSCLC	AIF	Silenced AIF strengthens glycolysis and sensitivity to glucose deprivation in KRAS G12D-driven lung cancer.	[48]
NSCLC	HK2	HK2 is required for KRAS G12D triggered lung cancer tumorigenesis.	[49]
NSCLC	NRF2	Glycolysis is the most significantly altered pathway between KRAS^G12D/G12D^ and KRAS^G12D/WT^, and NRF2 rewires glucose metabolism upon KRAS G12D copy number gain.	[50]
LUAD	TPI	LKB1 loss increases glycolysis flux via enhancing phosphorylation and stabilization of TPI1 in KRAS G12D-driven LUAD.	[51]
CRC	MYG1	KRAS mutated-induced MYG1 recruits PKM2 to phosphorylate PKM2 and sustain PKM2 in nucleus to active MYC downstream genes and glycolysis in colon cancer.	[52]
CRC	Vitamin C	Vitamin C uptake enhance ROS, and represses GAPDH to decrease glycolysis and ATP production ink KRAS mutant cancer.	[53]
CRC	Grp78	Grp78 heterozygosity reduces Apc-KRAS G12D driven CRC development via downregulation of GLUT1 levels and glycolysis.	[54]
PDAC, NSCLC, CRC	TCA flux	U-13C labelling lactate tracing profiling indicates KRAS-mutant tumor exerts suppressed TCA cycle fluxes and upregulated glycolysis.	[55]
GBM	PGK1	The KRAS G12V mutation triggers the translocation of PGK1 to the mitochondria, which promotes the phosphorylation and stabilization of PDHK1, leading to increased lactate production and facilitating brain tumorigenesis.	[56]
–	COXII	KRAS G12V leads to mitochondrial dysfunctions and induces HK2 expression.	[57]

*Abbreviations*

*HK1* hexokinase 1, *PDAC* pancreatic ductal adenocarcinoma, *COX-2* prostaglandin G/H synthase 2, *HFD* obesogenic high-fat diet, *GFPT1/2* glutamine-fructose-6-phosphate transaminase ½, *HK2* Hexokinase 2, *PFK1* phosphofructokinase-1, *LDHA* lactate Dehydrogenase A, *GM3* ganglioside 3, a sphingolipid, *SM4* 3-O-sulfogalactosylceramide, a sphingolipid, *JAK1/STAT6/MYC* JAK1, a key molecule in intracellular signaling pathways, activates STAT6, promotes MYC expression, regulates cell growth and immunity, *IL2Rγ* Interleukin-2 receptor gamma chain, *IL4R* Interleukin-4 Receptor, *PRMT1* protein arginine methyl transferase 1, *SLC2A1* solute carrier family 2, facilitated glucose transporter member 1, *5-HT* Serotonin, *Nutri-hijacker* biguanide-modified nanoparticulate albumin, *NOX4* NAD(P)H oxidase 4, *CA9* carbonic anhydrases 9, *NIX* NIP3-like protein X, *ATG7* Autophagy Related 7, *PAAD* pancreatic adenocarcinoma, *NSCLC* non-small cell lung cancer, *LIFR* cytokine leukemia inhibitory factor receptor, *STAT3* signal Transducer and Activator of Transcription 3, *FBP1* fructose-1,6-bisphosphatase, *AIF* apoptosis-inducing factor, *NRF2* nuclear Factor Erythroid 2-Related Factor 2, *LUAD* lung adenocarcinoma, *TPI* triosephosphate isomerase, *LKB1* serine/threonine kinase 11, *MYG1* melanocyte proliferating gene 1, *PKM2* pyruvate Kinase M2, *CRC* colorectal cancer, *ROS* Reactive Oxygen Species, *GAPDH* glyceraldehyde-3-phosphate dehydrogenase, *ATP* adenosine 5'-triphosphate, *Grp78* glucose-regulated protein 78, *GLUT1* facilitative glucose transporter 1, *TCA* tricarboxylic acid, *GBM* glioblastomas, *PGK1* phosphoglycerate kinase 1, *PDHK1* pyruvate dehydrogenase kinase, *COXII* cyclooxygenase-2

**Fig. 1:**
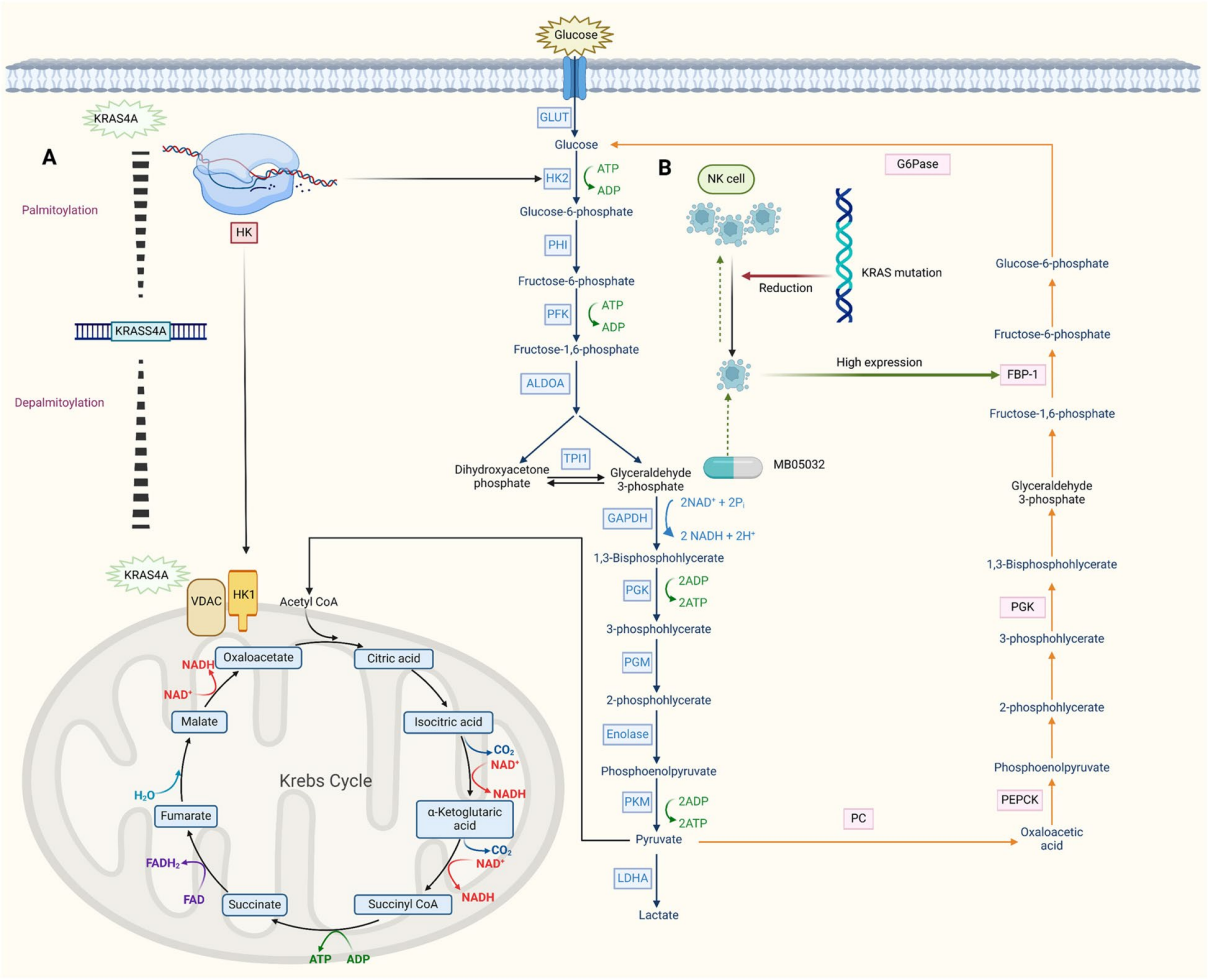
KRAS modulates glycolytic enzymes. **A** KRAS4A, one of two KRAS isoforms, interacts with hexokinase 1 (HK1) to enhance glycolytic flux. **B** Enforced FBP1 represses tumor glycolysis, whereas MB05032 molecular can restore this phenotype and promote tumor growth

**Fig. 2:**
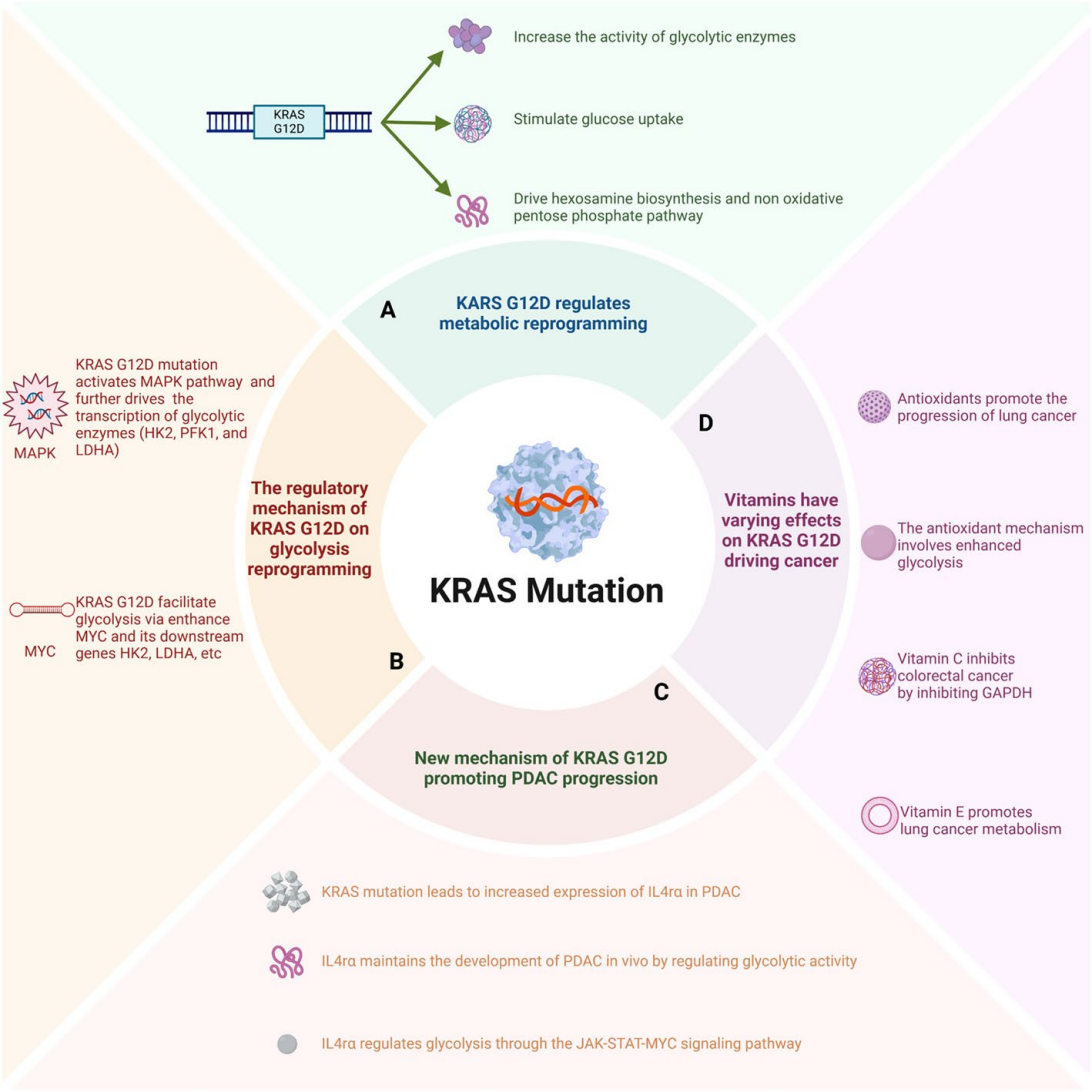
KRAS G12D mutation rewires glycolysis. **A** KRAS G12D mutations promote glycolysis process and drive hexosamine biosynthesis and the non-oxidative pentose phosphate pathway. **B** KRAS G12D regulates the glycolysis process through the MAPK and MYC signaling pathways. **C** KRAS G12D mutation increases glycolytic activity via IL4rα and JAK-STAT-MYC signaling in PDAC. **D** KRAS G12D influences glycolysis via vitamins

There are two distinct isoforms of KRAS (KRAS4A and KRAS4B), which differ in their C-terminal regions [58]. Amendola and colleagues reported that KRAS4A promotes glycolytic flux via interacting with hexokinase 1 (HK1), a key glycolytic enzyme located on the outer mitochondrial membrane (Fig. 1A) [34]. Ying found that withdrawal of KRAS G12D results in a significant reduction in glycolytic intermediates, including glucose-6-phosphate (G6P), fructose-6-phosphate (F6P), and fructose-1,6-bisphosphate (FBP). Moreover, KRAS G12D extinction also downregulates several rate-limiting glycolytic enzymes like HK1, hexokinase 2 (HK2), PFKl and lactate dehydrogenase A (LDHA), while decreasing the glucose uptake and lactate production in pancreatic cancer. In addition, they demonstrated that KRAS G12D modulates glycolytic reprogramming via MAPK and MYC pathways, stimulating glucose uptake and driving glucose intermediates into the hexosamine biosynthesis and nonoxidative pentose phosphate pathways (PPP) (Fig. 2A, B) [36]. Liu et al., explored this metabolic reprogramming in a KRAS^LSLG12D^/Tp53^fl/fl^ (KP) engineered mouse model with ARID1A loss (KPA). They observed that ARID1A loss in KPA mice accelerate lung tumor growth, and promote glycolysis activity, enhance the transcription of glycolytic intermediates such as PGAM1, PKM2, and PGK1 [59].

The Warburg effect plays a critical role in immune evasion [60, 61]. Cong performed a study using a CRE-inducible KRAS^LSLG12D^ mouse model, which revealed a significant reduction in NK cell population and an increase in glycolysis during various stages of KRAS-driven lung cancer. The study further identified that FBP1, an enzyme for glyconeogenesis, is upregulated in NK cells within lung tumors. Highly expressed FBP1 results in repressed glycolytic capacity, whereas inhibition of FBP1 with MB05032 sufficiently restores glycolysis and promotes tumor growth (Fig. 1B) [61, 47]. Additionally, KRAS mutation enhances the expression of Type I cytokine receptor IL4rα in pancreatic ductal adenocarcinoma (PDAC). IL4rα is required to maintain PDAC progression in vivo through a JAK-STAT-MYC modulated glycolytic activity. (Fig. 2C). Immunohistochemical (IHC) staining reveals that a substantial presence of T effector 1 (Th2) cells in tumor samples. Treatment with anti-CD4 Th2 neutralizing antibody leads to a significant reduction in tumor formation [38] .

Nutrition plays a significant role in cancer prevention, treatment, and recovery [62]. Wiel et al., reported that supplementation with the antioxidants N-acetylcysteine and vitamin E promotes the progression and metastasis of KRAS G12D-driven lung cancer. Mechanistically, these antioxidants stabilize HK2 and glyceraldehyde 3-phosphate dehydrogenase (GAPDH) expression, enhance glucose uptake, increase glycolytic rates and stimulate lactate secretion, thereby facilitating glycolysis-responsive lung cancer development [63]. In contrast, vitamin C, another member of vitamin family, inhibits KRAS G12D-driven colorectal cancer. Specifically, vitamin C represses glycolysis and inactivates GAPDH, a key rate-limiting glycolytic enzymes. This inhibitory effect can be counteracted by either inhibiting PARP or supplementing with nicotinamide mononucleotide (Fig. 2D) [53].

### KRAS and lipid metabolism

Lipid metabolism is a complex and tightly regulated processes which is crucial for energy storage, membrane structure, and cellular signaling [64]. Cancer cells often reprogram their lipid metabolism to support rapid proliferation and energy production. This reprogramming involves lipid synthesis, alterations in fatty acid oxidation, lipid droplet formation, and modulation of lipid signaling pathways [22]. The study of lipid metabolism has gained significant attention, particularly regarding its interplay with KRAS mutations in cancer (Table 2) [65]. Hereby, we outline key points on how KRAS mutations influence lipid metabolism.

**Table 2:** KRAS and Lipid metabolism in cancer

Cancer type	Target	Function and Mechanism	Ref
Pan-cancer	SG	KRAS elevates 15-d-PGJ2-dependent SG, rendering resistance to stress stimuli.	[66]
PDAC	ACLY	ACLY produces nuclear cytoplasmic acetyl coenzyme A, which facilitates histone acetylation and cholesterol biosynthesis during ADM of PDAC.	[67]
PDAC	NSDHL	Cholesterol biosynthesis induces NSDHL to repress TGF-β-induced EMT and PDAC development.	[68]
PDAC	SLC25A1	KRAS G12D recruits GLI1 to enhances SLC25A1, therefore activating fatty acid uptake.	[69]
NSCLC/PDAC	ACSL3	Mutant KRAS promotes cellular uptake, retention, accumulation, and β-oxidation of fatty acids in lung cancer in an ACSL3-dependent manner; ACSCL3 loss promotes autophagy and suppresses xenografted of PDAC.	[70, 71]
NSCLC	ACC	ACC inhibitor ND-646 suppresses fatty acid synthesis in vivo and in vitro of KRAS G12D driven lung cancer.	[72]
PAAD/LUAD	REDD1	REDD1 loss triggers HIF-dependent activation of lipid storage pathways, involving PPARγ and CD36, resulting in KRAS mutant PDAC initiation and poor outcomes.	[73]
LUAD	FASN	KRAS mutant induces de novo lipogenesis via FASN, while silenced FASN induces accumulation of PUFA-phospholipids and ferroptosis in lung cancer.	[74]
CRC	AMPK	Adipocytes induce autophagy, alter cellular metabolism, support cell survival upon nutrient deprivation and prevent cell differentiation in colon cancer cells.	[75]
CRC	n-3PUFA	Long-chain n-3PUFA reshapes KRAS nanoscale proteolipid by reducing the lateral segregation of cholesterol-dependent and -independent nanoclusters, thereby attenuating oncogenic KRAS-mediated ERK signaling.	[76]
CRC	TFCP2	KRAS mutation triggers lipid-rich CAF via activation of TFCP2/BMP4/WNT5B/VEGFA axis.	[77]
BRCA	mTORC1	KRAS G12V induced mTORC1 and SREBP drives de novo lipid synthesis to promote aberrant growth in breast cancer.	[78]
Gastric cancer	SCD	KRAS G12D-induced SCD fuels dysplastic cell hyperproliferation and facilitates high grade dysplasia in gastric cancer.	[79]
–	IFNGR1	Sphingolipids dampen cytotoxic immunity, while deletion of sphingolipid sensitizes cancer cells to IFNγ signalling via increases surface levels of IFNGR1.	[80]

*Abbreviations*

*SG* stress granules, *PDAC* pancreatic ductal adenocarcinoma, *ACLY* ATP-citrate lyase, *ADM* acinar-to-ductal metaplasia, *NSDHL* NAD(P) Dependent Steroid Dehydrogenase-Like, *EMT* epithelial-mesenchymal transition, *SLC25A1* solute carrier family 25 member 1, *GLI1* glioma-associated oncogene homolog 1, *NSCLC* non-small cell lung cancer, *ACSL3* Acyl-coenzyme A (CoA) synthetase long-chain family member 3, *ACC* acetyl-CoA carboxylase, *PAAD* pancreatic adenocarcinoma, *LUAD* lung adenocarcinoma, *REDD1* regulated in development and DNA damage responses 1, *HIF* hypoxia-inducible factor, *PPARγ* peroxisome proliferatorsactivated receptors, *CD36* cluster of differentiation 36, *FASN* fatty acid synthase, *CRC* colorectal cancer, *AMPK* AMP-activated protein kinase, *n-3PUFA* long-chain n-3 polyunsaturated fatty acids, the first unsaturated bond in polyunsaturated fatty acids occurs at the third position of the methyl end of the carbon chain, *ERK* extracellular regulated protein kinases, *TFCP2* transcription factor CP2, *BMP4* Bone Morphogenetic Protein 4, *WNT5B* Wnt Family Member 5B, *VEGFA* Vascular endothelial growth factor A, *BRCA* breast cancer, *mTORC1* mechanistic target of rapamycin complex 1, *SREBP* sterol regulatory element-binding proteins 1, *SCD* stearoyl- coenzyme A desaturase, *IFNGR1* IFNγ receptor subunit 1

**Fig. 3:**
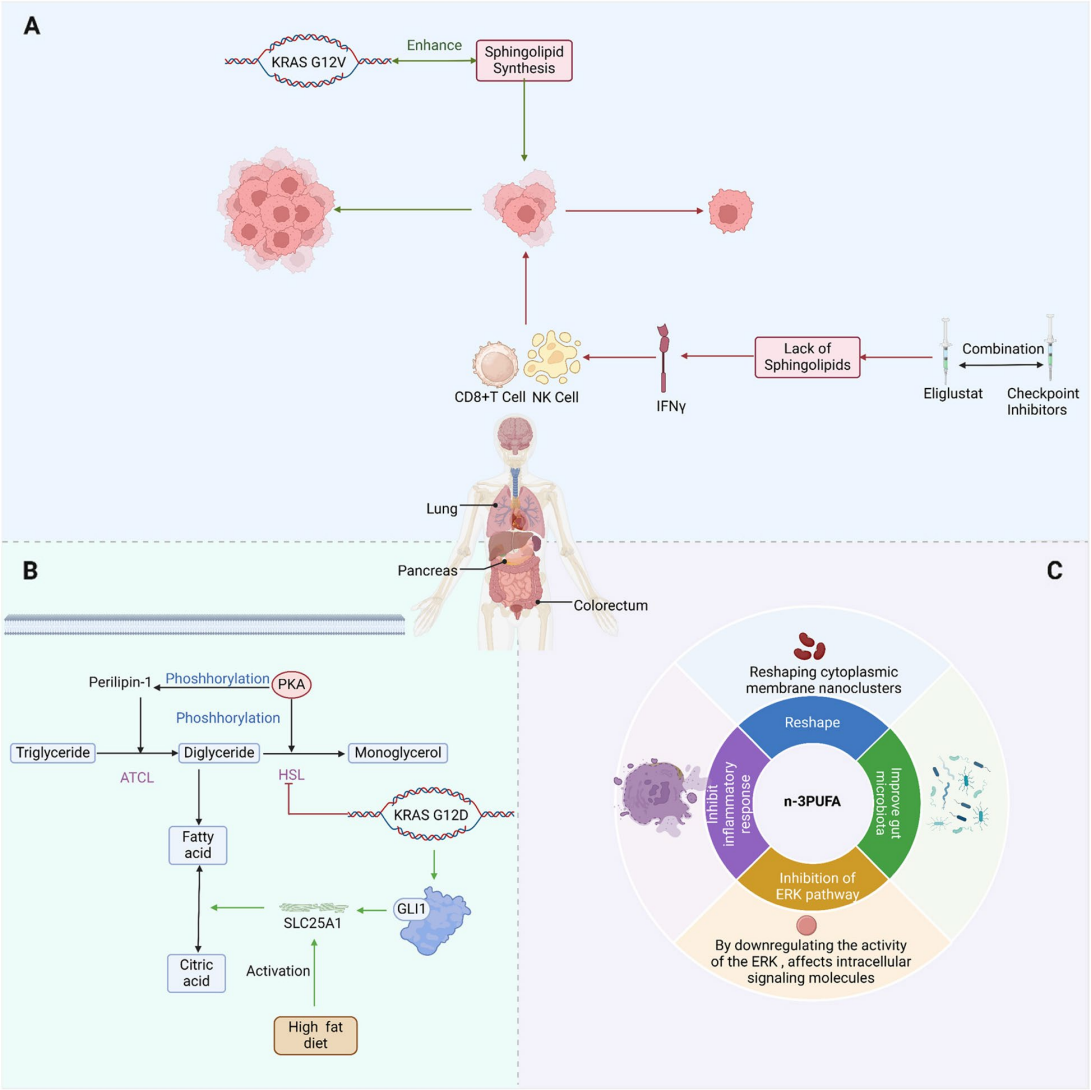
KRAS and lipid metabolism. **A** Sphingolipid deficiency enhances the pro-apoptotic effect of natural killer cells (NK cells) and CD8 + T cells by activating the interferon gamma signaling in KRAS G12V-driven lung cancer. **B** KRAS G12D enhances lipid droplet utilization and storage via HSL, SLC25A1 and GLI1 in PDAC, respectively. **C** Supplementing with n-3 PUFA can downregulate ERK signalling, improve gut microbiota and reshape tumor microenvironment

Sphingolipid synthesis is essential for cancer proliferation [81]. Soula recently showed that sphingolipid deficiency enhances pro-apoptotic effect of NK cells and CD8 + T cells via the activation of interferon-γ (IFNγ) signalling. The loss of sphingolipid synthesis sensitizes cancer cells to IFNγ treatment. Clinically, combining eliglustat, a glycosphingolipids inhibitor, with checkpoint blockade therapies such as anti-CTAL4 or anti-PD-1, has been shown to suppress KRAS-driven lung adenocarcinoma (LUAD) [80]. In addition, Liu and colleagues reported that KRAS G12V enhance the synthesis of glycosphingolipids. They also found that glycosphingolipid synthesis is required for KRAS functions, indicating KRAS and glycosphingolipid synthesis form a reciprocal axis in cancer (Fig. 3A) [37].

Rozeveld et al., reported that excess lipid promotes PDAC invasion and migration through lipolysis. KRAS G12D enhances this utilization and storage of lipid droplets by downregulating hormone-sensitive lipase (HSL). In addition, enforced HSL expression converts metabolism from glycolysis to oxidative phosphorylation in KRAS G12D-driven PDAC (Fig. 3B, Top) [82, 83]. KRAS G12V promotes steatosis and prevents hyperplasia in hepatocellular carcinoma (HCC). However, Wnt/MYC signalling can attenuate the extensive hepatic steatosis and senescence triggered by KRAS expression. MK8245, an inhibitor of stearoyl-CoA desaturase (SCD), strikingly inhibits hepatomegaly in Tg (Wnt&KRAS) and Tg (MYC & KRAS) mouse models, implying fatty acid desaturation is an important therapeutic target for antagonizing the combined effects of Wnt and RAS signaling pathways in HCC [84]. Fuentes et al., demonstrated that dietary supplemented with fish oil containing long-chain n-3 polyunsaturated fatty acids (n-3 PUFA), such as eicosapentaenoic acid (EPA) and docosahexaenoic acid (DHA), reduces CRC in an inducible genetic model. This effect is attributed to the reshaping of cholesterol-dependent and -independent nanoclusters and downregulation of ERK signaling (Fig. 3C) [76]. KRAS can enhance fatty acid uptake, fatty acid oxidation (FAO), and lipogenesis in pancreas via GLI1-induced SLC25A1, an important citrate transporter which plays a key role in FAO and fatty acid synthesis in PDAC. A high-fat diet (HFD) further stimulates the KRAS G12D-GLI1-SLC25A1 axis, leading to increased citrate and fatty acids. Pharmacological targeting GLI1 or SLC25A1 could suppress KRAS G12D-driven PDAC in the context of a HFD (Fig. 3B, Bottom) [69]. Acetyl-CoA carboxylase (ACC) control fatty acid synthesis [85]. Svensson reported that chronic treatment with ND-646 (ACC inhibitor) remarkedly dampens KRAS-driven lung cancer [72].

### KRAS and amino acid metabolism

Amino acid metabolism is vital for protein synthesis, energy production, and the synthesis of various bioactive molecules [86]. This process is tightly regulated to meet the physiological demands of the body, and its dysregulation is associated with various diseases, including metabolic disorders and cancer [87, 88]. KRAS also regulates these metabolic pathways (Table 3). Targeting the metabolic vulnerabilities offers a promising approach to developing effective therapies against KRAS-driven cancers.

**Table 3:** KRAS and amino acid metabolism in cancer

Cancer type	Targeting	Function and Mechanism	Ref
PDAC	OAT	KRAS drives DNS and polyamine synthesis via induction of OAT, leading to alterations in the transcriptome and open chromatin landscape in PDAC cells.	[89]
PDAC	DON	DON and DRP-104 reduce oxidative phosphorylation, glycolysis and TCA cycle, which resulting in inhibiting PDAC tumor development.	[90]
PDAC	MDH1	Depletion of O-GlcNAcylation reduces MDH1 activity, impairs glutamine metabolism and sensitizes KRAS mutant PDAC cells to oxidative stress.	[91]
PDAC	BCAA, BCAT2	BCAT2 is essential for KRAS G12D PDAC by increasing BCAA uptake.	[92]
PDAC, CRC	–	Restriction of dietary serine and glycine reduces tumour growth in xenograft and allograft models, however, KRAS G12D confers resistance to the anti-cancer effects.	[93]
CRC	SLC25A21	KRAS mutant promotes CRC tumorigenesis by downregulation of SLC25A21 and αketoglutarate.	[94]
CRC	SLC25A22	SLC25A22-mediated glutamine metabolism enhances CXCL1 chemokines level via asparagine biosynthesis to promote KRAS-mutant CRC.	[95]
CRC	YAP1	Oncogenic KRAS mutations enhance AAT expression through the hippo effector YAP1, leading to mTOR activation and CRC cell proliferation.	[96]
NSCLC	SLC7A1	Inhibition of ASS1 in KRAS mutant NSCLC cells affects arginine biosynthesis, resulting in a dependence of extracellular arginine input on the arginine transmembrane transporter protein SLC7A1.	[97]
NSCLC	ATF4	NRF2-mediated ATF4 contributes KRAS-induced amino acid transport and metabolism.	[98]
LUAD	STAT3	pS727-STAT3 enhance KRAS-induced LUAD development by impairing mitochondrial metabolism.	[99]
LUAD	SLC7A11	The SLC7A11 inhibitor HG106 suppresses cystine uptake and intracellular glutathione biosynthesis, impairing tumor growth and promoting beneficial outcome.	[100]
LUAD	NRF2	NRF2/KEAP1 and LKB1 loss cooperatively rewire metabolism and enhance glutaminase inhibitor CB-839 sensitivity in lung cancer.	[101]
–	–	Synergy between LKB1 loss and KRAS mutations enhances de novo serine biosynthesis and supports tumor growths.	[102]

*Abbreviations*

*PDAC* pancreatic ductal adenocarcinoma, *OAT* ornithine aminotransferase, *ODC1* ornithine decarboxylase 1, *DON* 6-diazo-5-oxoL-norleucine, *DRP-104* sirpiglenastat, *MDH1* Malate dehydrogenase 1, *BCAA* branched-chain amino acid, *BCAT2* BCAA transaminase 2, *O-GlcNAcylation* O-linked β-N-acetylglucosamine, *CRC* colorectal cancer, *SLC25A21* solute carrier family 25 member 21, *SLC25A22* solute carrier family 25 member 22, *YAP1* yes1 associated transcriptional regulator, *AAT* amino acid transporters (SLC7A5/LAT1, SLC38A2/SNAT2, and SLC1A5/ASCT2), *mTOR* mechanistic target of rapamycin, *NSCLC* non-small cell lung cancer, *SLC7A1* solute carrier family 7 member 1, *ASS1* arginine succinate synthase 1, *ATF4* activating transcription factor 4, *NRF2* nuclear factor erythroid 2-related factor 2, *LUAD* lung adenocarcinoma, *STAT3* signal transducer and activator of transcription 3, *SLC7A11* solute carrier family 7 member 11, *KEAP1* Kelch Like ECH Associated Protein 1, *LKB1* liver kinase B1

**Fig. 4:**
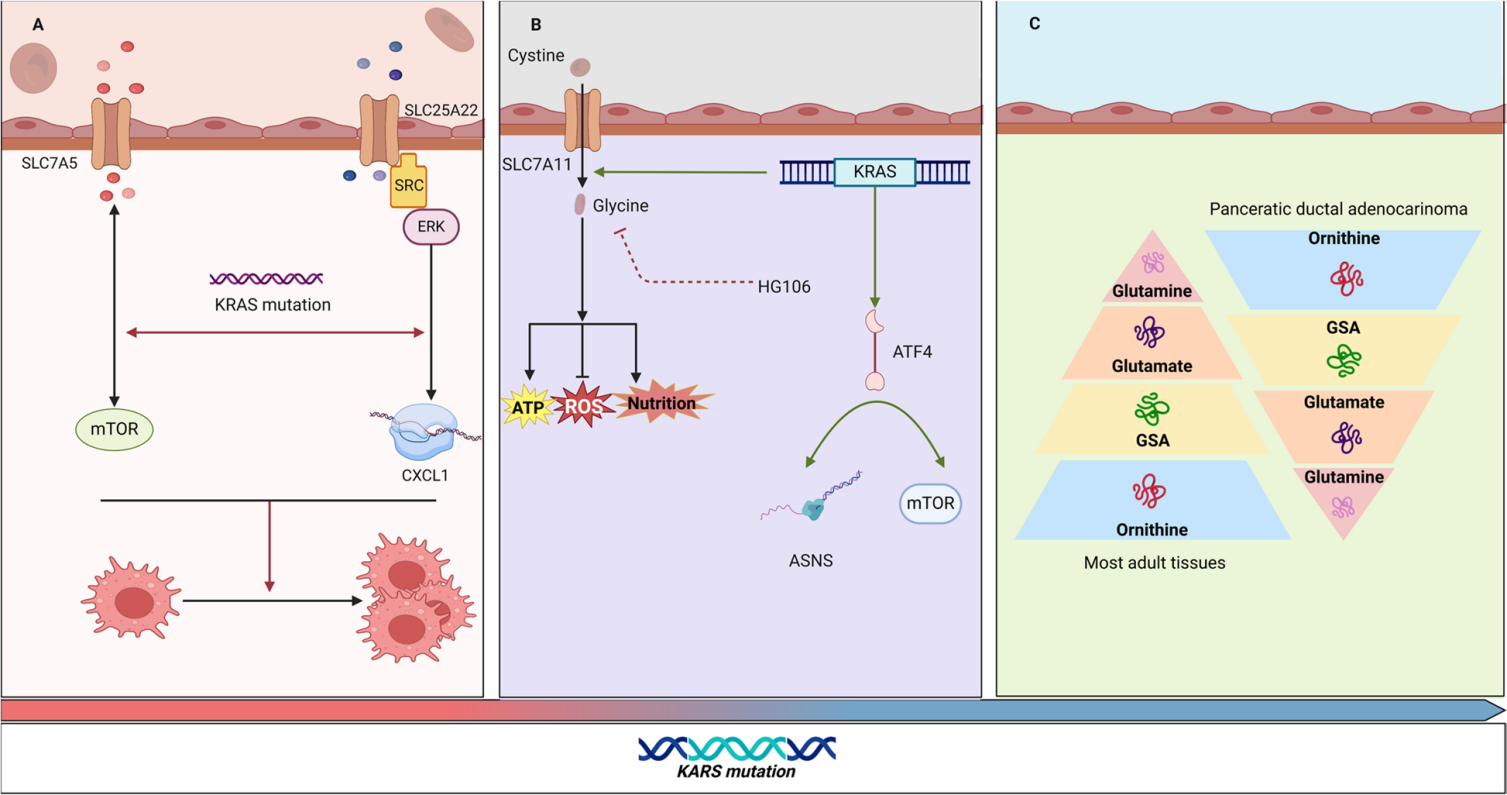
KRAS modulates amino acid metabolism. **A** KRAS promotes CRC via SLC7A5 and mTOR signalling in colon cancer. **B** SLC7A11 and ATF4 are essential for KRAS-modulated lung cancer. **C** KRAS triggers de novo ornithine synthesis in PDAC

Najumudeen observed that KRAS rewires the amino acid metabolism in colorectal cancer (CRC) tumorigenesis through its dependency on SLC7A5, a glutamine antiporter. Mechanistically, SLC7A5 maintains intracellular amino acid levels, and its loss suppresses mTOR signaling. The abrogation of SLC7A5, in conjunction with mTORC1 inhibition, represses KRAS-driven CRC development (Fig. 4A, Left) [103]. In addition, SLC25A22 drives asparagine uptake, which binds and activates SRC phosphorylation. SRC subsequently recruits ERK, facilitating the transcription of CXCL1 (Fig. 4A, Right). Targeting SLC25A22 in combination with anti-PD1 therapy synergizes with immunological surveillance to suppress KRAS-mutant CRC growth in vivo [95]. SLC7A11, another glutamate antiporter, is required for KRAS-mutant driven lung cancer. Inhibition of SLC7A11 by HG106 decreases cystine uptake, enhances cytotoxicity, and promotes oxidative stress- and ER stress-mediated cell apoptosis (Fig. 4B, Left) [100]. Dietary restriction with low methionine has been shown to inhibit KRAS-driven CRC and increase sensitize to radiation therapy [104]. Lee demonstrated that PDAC exerts a distinct dependence on de novo ornithine synthesis from glutamine. KRAS mutant drive uptake of arginine-derived ornithine, induce expression of ornithine aminotransferase (OAT), and eventually promote PDAC development (Fig. 4C) [89].

Several studies have highlighted the essential role of asparagine synthetase (ASNS) in tumorigenesis [105–107]. For example, Lin showed that knockout of autophagy gene ATG5 impairs KRAS G12V-driven tumor growth. Metabolite profiling revealed a deficiency in the nonessential amino acid asparagine alongside overexpressed ASNS. Inhibition of ATG5 and ASNS represses cell proliferation, migration, and invasion [108]. KRAS can enhance asparagine biosynthesis via ATF4 in NSCLC. ATF4 activates ASNS, thus repressing apoptosis, facilitating protein biosynthesis, and activating mTORC1 pathway (Fig. 4B, Right [98]) .

### KRAS and glutamine metabolism

Glutamine, the most abundant amino acid in the bloodstream, serves as an important metabolic fuel for rapidly proliferating cells. It supports the increased demand for ATP, biosynthetic precursors, and reducing agents [24]. In cancer cells, glutamine is transported into cell through the amino acid transporter ASCT2/SLC1A5. It then undergoes a catabolized process called glutaminolysis, where it is converted into glutamate and alpha-ketoglutarate (α-KG), an intermediate of the tricarboxylic acid (TCA) cycle which is essential for energy production and biosynthesis [109].

KRAS also alters glutaminolysis [110]. Wong and colleagues observed increased glutamine uptake and a reduced α-KG composition in KRAS-mutant CRC. They also found a reduced DNA demethylation and hypermethylation at CpG sites of WNT signalling genes. Furthermore, SLC25A22 is essential for the KRAS mediated DNA methylation, while silencing SLC25A22 activates H3K4me3 demethylation in promoter regions of stemness-associated genes, and suppresses LGR5 expression in CRC [111]. 6-diazo-5-oxo-L-norleucine (DON), a glutamine (Gln) antagonist, can lead to a metabolic crisis. Similarly, sirpiglenastat (DRP-104), a pro-drug version of DON, reduces tumor growth in KRAS driven-PDAC [112]. Hu employed a genetic model harboring KRAS G12D activation and SIRT5 inhibition, finding that SIRT5 loss enhances glutamine and glutathione metabolism via glutamate–oxaloacetate transaminase 1 (GOT1), and eventually promoting pancreatic tumorigenesis [113]. In addition, mitochondrial uncoupling protein 2 (UCP2) can also trigger glutamine metabolism. Silencing UCP2 in KRAS mutant cells decreases glutaminolysis, cell proliferation, and tumor growth in PDAC [114].

In summary, cancer metabolism involves a complex and dynamic reprogramming of cellular metabolic pathways, allowing cancer cells to thrive in challenging environments. These metabolic adaptations provide potential targets for therapeutic intervention, offering new strategies to disrupt the metabolic dependencies of tumors.

## KRAS and tumor microenvironment

The tumor microenvironment encompasses the complex and dynamic surroundings of a tumor, including various cell types, extracellular matrix components, signaling molecules, and blood vessels [115, 116]. Metabolic reprogramming within the tumor microenvironment supports the high biogenesis demand of cancer cells for rapid proliferation, and helps tumor cell survive under the genetic or environmental stresses [117]. Emerging studies have shown that tumor microenvironment and cancer metabolism are deeply intertwined, with each influencing and shaping the other [118, 119]. KRAS mutations play a significant role in shaping the tumor microenvironment and influencing cancer progression [120, 121]. The following section provides a detailed examination of how KRAS mutations interact with and impact the tumor microenvironment via immune cells, chemokines and cytokines.

### KRAS and immune cells

**Fig. 5:**
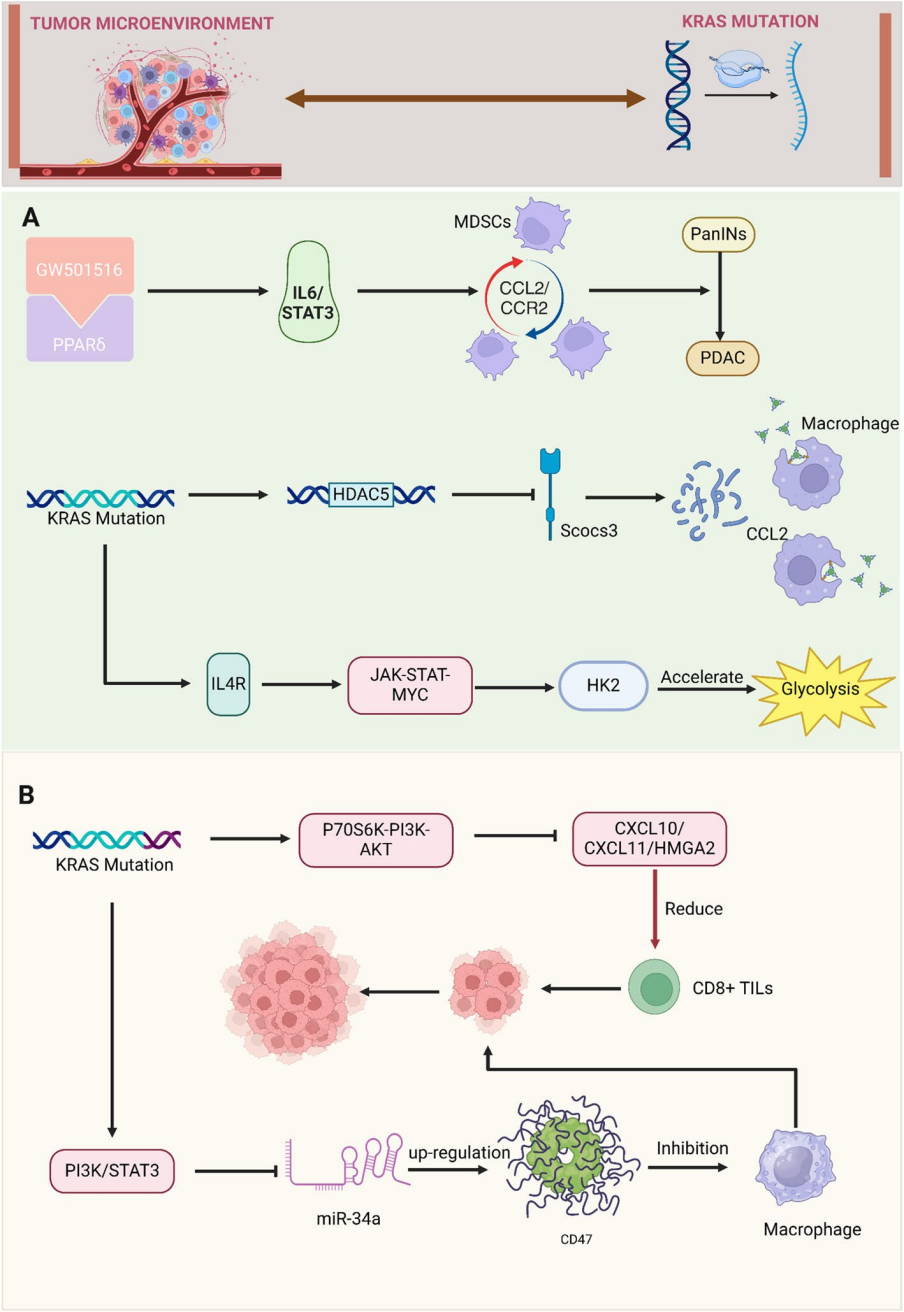
KRAS and tumor microenvironment. **A** Top: HPD and its agonist GW501516 alter the tumor microenvironment by recruiting macrophages and myeloid-derived suppressor in KRAS mutated PDAC. Bottom: KRAS mutation promote the occurrence of PDAC via HDAC5/CCL2 and IL4R/JAK-STAT-MYC pathways. **B** KRAS G12D activate P70S6K/PI3K/AKT signaling, and further represses CXCL10/CXCL11 and microRNA-34a, ultimately inhibiting macrophage and CD8 + tumor infiltrating lymphocytes in lung cancer

The TME is a complex and dynamic environment composed of cancer cells, stromal cells, blood vessels, immune cells, and signaling molecules. Immune cells within the TME can either help eliminate cancer cells or, conversely, contribute to tumor growth and immune evasion [122]. Mutational alterations in oncogenes like KRAS influence the inflammatory status of tumors and affect immune elimination, even during immunotherapy or vaccination strategies [123]. Liu reported that pancreatic intraepithelial neoplasia lesions (PanINs) frequently harbor KRAS mutations but rarely progress to PDAC. However, activation of the peroxisome proliferator-activated receptor-delta (PPARδ) ligand by High fat diets (HFD) or its agonist GW501516 enhances IL6/STAT3 signalling and rewires PDAC tumor microenvironment. This alteration is mediated through the induction of CCL2 secretion, which recruits immunosuppressive macrophages and myeloid-derived suppressor cells into the pancreas via the CCL2/CCR2 axis. This recruitment orchestrates an immunosuppressive tumor microenvironment and drives the progression of PanINs to PDAC (Fig. 5A, Top) [121]. Moreover, tumor-associated neutrophils (TANs) affect cell fate and tumor microenvironment of lung cancer. Koyama et al., deciphered that genetic silencing of STK11/LKB1 in KRAS-driven NSCLC results in the accumulation of neutrophils and T-cell exhaustion [124]. In the KRAS-driven lung squamous cancer, enforced expression of SOX2 and/or loss of NKX2-1 leads to an increased TANs and promotes lung cancer tumorigenesis. The authors performed a ChIP-seq and observed that increased TANs are required for SOX2-induced transcription of chemokine CXCL5, a well-known neutrophil chemoattractant gene [125]. Ottaiano and colleagues observed that CD3^+^/CD8^+^ lymphocytes derived from oligometastatic CRC patients exhibit distinct cytotoxic activity against human colon cancer cells harboring KRAS mutations [126]. In addition, they demonstrated that the loss of KRAS mutations in lung-specific oligometastatic CRC further suggests a unique evolutionary trajectory, potentially linked to reduced aggressiveness or changes in the tumor microenvironment [127]. Hosein generated a generated Ptf1a-Cre;LSL-KRAS G12D;Rnf43flox/flox (KRC) and Ptf1a-Cre; LSL-KRAS G12D (KC) mice and found that KPC mice with lack RNF43, exert poor survival, an increase in T and B lymphocytes, as well as a decrease macrophages. They further found that CXCL5 is decreased in KPC mice, compared to KC mice, which may responsible for the tumor formation in PDAC [128].

### KRAS and chemokines

Chemokines are a family of small signaling proteins that play a crucial role in the immune system by directing the migration, activation, and positioning of immune cells. They are essential for immune surveillance, inflammation, tissue repair, and cancer progression [129, 130]. Hou et al., identified HDAC5 is a potential downstream target of KRAS via an iKPC mice model. Enforced HDAC5 represses Socs3, thus upregulating CCL2 expression and promoting CCL2-recruited macrophages. These tumor-associated macrophages (TAMs) provide cancer cells with trophic support, including TGFβ/SMAD4, enabling KRAS mutation to bypass inhibitory mechanisms in PDAC development (Fig. 5A, Middle) [131]. KRAS G12D mutant also affect immune landscape in NSCLC by repressing CD8^+^ tumor-infiltrating lymphocytes (TILs) through inhibition of chemokines CXCL10/CXCL11 in NSCLC. Mechanistically, KRAS G12D actives P70S6K/PI3K/AKT signalling and reduces CXCL10/CXCL11/HMGA2 level. In comparison to PD-L1 inhibitor monotherapy, chemo-immunotherapy of PD-L1 blockade with paclitaxel significantly suppressed tumor growth in a mouse model of KRAS G12D-mutant lung adenocarcinoma (Fig. 5B, Top) [132]. Liao demonstrated that the KRAS-IRF2-CXCL3-CXCR2 axis is a potential target for therapeutic strategies in CRC, particularly in tumors resistant to current ICB therapies. Specifically, oncogenic KRAS represses IRF2 expression, leading to elevated CXCL3 expression, a chemokine that recruits myeloid-derived suppressor cells (MDSCs) into the TME. Increased MDSC infiltration suppresses cytotoxic T cell responses, creating an immune-suppressive environment. Restoring IRF2 expression or inhibiting CXCR2, a CXCL3 receptor, reduces MDSC infiltration and enhances T cell-mediated anti-tumor responses in colorectal cancer [133].

### KRAS and cytokines

Cytokines are small proteins secreted by cells, especially immune cells, that act as signaling molecules to regulate and coordinate immune responses. They play a crucial role in inflammation, immunity, and tissue repair [134, 135]. Dey and colleagues identified that IL4R is among the top 50 most overexpressed genes in KRAS-driven PDAC tumors. IL4R can promote PDAC in vitro and in vivo via activating of JAK-STAT-MYC signalling, which enhances glycolysis by increasing HK2 expression and extracellular acidification rate (ECAR) (Fig. 5A, Bottom). Utilizing the Pdx-Cre;KRAS^LSLG12D^ model, the authors detected enriched IL4, STAT6 and MYC level in these tumor lesion, which are dramatically infiltrated by CD4 + T cells, potentially contributing to pancreatic cancer initiation and progression [38]. Jang et al., reported that Leukotriene B4 receptor-2 (BLT2) plays a pivotal role in KRAS-driven lung tumor progression by promoting IL-6-mediated inflammation. High BLT2 expression is observed in human lung adenocarcinomas with KRAS mutations, whereas BLT2 inhibition, either pharmacologically or through genetic knockout, decreases lung tumor formation, IL-6 production, and airway inflammation in KRAS-mutant mouse models [136]. Oncogenic KRAS G12D in PDAC cells upregulates IL-33, a cytokine that recruits and activates type 2 immune cells like T_H_2 and ILC2. These immune cells secrete cytokines like IL-4 and IL-13, which drive tumor progression through immune suppression and metabolic reprogramming of cancer cells [137].Interestingly, Donahue and colleagues demonstrated that stromal IL-33 production by cancer-associated fibroblasts (CAFs) is driven by signals from epithelial cells harboring the KRAS G12D mutation. This process supports a pro-tumorigenic environment by promoting immunosuppressive changes and reducing tumor-infiltrating CD8 + T cells. Targeted deletion of IL33 in stromal cells using compartment-specific knockout mouse models results in increased CD8 + T-cell infiltration and activation, reprogramming of the tumor microenvironment-including CAFs and myeloid cells-toward a less immunosuppressive state, and a reduction in tumor growth [138]. Hu depicted that KRAS mutation impairs the macrophage phagocytosis sensitivity by increasing CD47 expression in lung cancer. Mutant KRAS enhances PI3K/STAT3 signalling, which in turn, represses microRNA-34a, leading to increased CD47 expression. Therapeutical targeting KRAS, STAT3 or re-overexpressed microRNA-34a can restore the inmate immune surveillance and favor a beneficial therapy (Fig. 5B, Bottom) [139].

### KRAS and tumor heterogeneity

Cancers exhibit substantial heterogeneity, meaning that individual cancer cells within the same tumor mass can have distinct metabolic and genetic phenotypes [140]. Even tumors of similar molecular profiles can vary significantly between patients in terms of genetic mutations, molecular signatures, and treatment responses. This variability contributes to differences in clinical outcomes and therapeutic effectiveness. For instance, KRAS G12D activation and p53 deletion foster both NSCLC and PDAC development. However, these tumors exert distinct patterns of amino acid metabolism. NSCLC is characterized by increased uptake of branched-chain amino acids (BCAAs) and elevated activity of BCAA catabolic enzymes, whereas PDAC tends to inhibit BCAAs uptake. The removal of Bcat1 and Bcat2, the enzymes responsible for BCAA catabolism, renders NSCLC formation, but do not affect PDAC tumorigenesis [141]. These findings underscore the heterogeneity of cell of origin and tissue microenvironment in influencing genetic events and metabolic reprograms.

## KRAS and epigenetic modification

Epigenetic modifications significantly influence cancer metabolism by altering the expression of genes involved in metabolic pathways. These changes can impact how cancer cells acquire and utilize nutrients, adapt to the tumor microenvironment, and respond to treatments [142, 143]. Notably, KRAS mutations not only drive oncogenic signaling but also lead to substantial alterations in the epigenetic landscape of cancer cells. Understanding these interactions is essential for developing new therapeutic strategies that target both the genetic and epigenetic drivers of cancer.

PDAC is a lethal cancer characterized by co-occurring oncogenic KRAS mutation and inactivating TP53 mutations. However, recapitulated p53 in KRAS G12D mutant PDAC increases the levels of α-KG, an essential metabolite that serving as substrate for chromatin modifying enzymes. ATAC-seq and RNA-seq analyses reveal that α-KG treatment can mimic the effect of p53 on global chromatin occupancy and gene regulation. The loss of p53 during PDAC development is associated with decreased levels of 5-hydroxymethylcytosine (5hmC); however, reactivation of p53 or α-KG treatment can restore 5hmC levels. Conversely, succinate, which represses α-KG, renders p53-driven tumor suppression. These findings suggest that an increased αKG/succinate ratio appears to be important for the modifying chromatin marks associated with premalignant cell fate, and contributes functionally to p53-driven tumour suppression [144]. In addition, another study presented that methyl cytosine-guanine dinucleotide (CpG) binding protein 2 (MECP2) is frequently amplified and overexpressed in various human cancers. MCEP2 functions similarly to activated KRAS across different tumor types via its DNA binding access to 5hmC [145].

DNA methylation is a key epigenetic modification that regulates gene expression and maintains genomic stability [146]. Mutant KRAS induces aberrant DNA methylation patterns, silencing tumor suppressor genes and activation of oncogenes, thereby contributing to cancer progression [147]. Luo found that high-frequency methylation adenoma cohorts also harboring KRAS mutation in CRC [148]. The nuclear factor of activated T cells 1 (NFATC1) is rapidly induced in response to acinar cell injury in both human and murine pancreatic cells and tissues. However, it is repressed by enhancer of zeste homologue 2 (EZH2)-dependent histone methylation, which in turn, facilitates acinar cell redifferentiation. Surprisingly, EZH2 depletion in KRAS G12D-driven PDAC resultes in a significant reduction of NFATC1 expression, suggesting KRAS serves as a regulatory switch modifying EZH2 responsive function on NFATC1 expression [149]. Additionally, N6-methyladenosine (m^6^A) in mRNA is another crucial epigenetic modification influencing cancer self-renewal and progression [150]. In lung cancer, KRAS mutations alongside loss of the tumor suppressor gene LKB1 are significantly correlated with m^6^A modifications, particularly affecting AlkB family member 5 (ALKBH5) levels. LKB1 loss results in high expression of ALKBH5 due to hypermethylation of CTCF-binding motif on ALKBH5 gene promoter, which prevents CTCF binding but fosters histone modification of H3K4me3, H3K9ac, and H3K27ac. As a result, this stabilizes several oncogenic drivers including SOX2, MYC and SMAD7 in lung cancer [151]. Moreover, Kottakis conducted gene set enrichment analysis (GSEA) of RNA-sequencing (RNA-seq) and quantitative proteomics to examine the synergy between LKB1 loss and KRAS mutation, revealing a close association with glycolysis. These KL cells exhibit increased glucose uptake, and elevation of GLUT1 and ATP production. In addition, LKB1 inactivation, in conjunction with KRAS G12D mutation, promotes serine biosynthesis, contributing to DNA methylation and the enrichment at retrotransposon elements associated with their transcriptional silencing [102].

Over the past two decades, extensive research has highlighted the versatile roles of non-coding RNAs (ncRNAs) in cancer. ncRNAs are a diverse group of RNA molecules that do not encode proteins but play crucial roles in regulating gene expression and various cellular processes [152, 153]. Many ncRNAs, particularly long noncoding RNAs (lncRNAs), are predominately localized in the nucleus, where they participate in chromatin modification, transcription, and different nuclear condensates [154]. microRNA-30a is downregulated in KRAS mutant CRC. It directly targets malic enzyme 1 (ME1) and inhibits cell proliferation via downregulation of NADPH production and fatty acid synthesis, thereby affecting KRAS-mutant-driven CRC progression [155]. Oncogenic lncRNAs such as H19 and metastasis-associated lung adenocarcinoma transcript 1 (MALAT1) interact with the KRAS signaling network [156]. H19 influences metabolic reprogramming by affecting the glycolytic enzymes such as 6-phosphofructo-2-kinase/fructose-2,6-biphosphatase 3 (PFKFB3) and LDHA, thereby altering glycolysis in oral and gastric cancers [157, 158]. MALAT1 stimulates glucose uptake, upregulates GLUT1, HK2, ENO1 and PKM2, and represses gluconeogenesis via TCF7L2 in HCC [159]. Additionally, exosomal MALAT1 from M2 TAMs reinforces aerobic glycolysis and tumorigenesis by stabilizing δ-Catenin protein and upregulating HIF-1α in gastric cancer [160]. Moreover, MALAT1 knockdown results in reduced levels of genes involved in fatty acid biosynthesis in HCC [161].

## KRAS targeted therapy

KRAS has historically been deemed "undruggable" due to the challenges by directly targeting the GTP binding pocket [162]. However, recent advances have led to the development of therapies for KRAS-driven cancers. These advancements encompass both direct inhibitors of KRAS and strategies aimed at targeting downstream signaling pathways and associated vulnerabilities (Table 4).

**Table 4:** KRAS therapy in cancer

Drug	Developer	Condition
**KARS G12C**		
Sotorasib (AMG 510)	Amgen	First FDA approved KRAS G12C inhibitor for NSCLC.
Adagrasib (MRTX849)	Mirati	FDA approved inhibitor for patients with advanced NSCLC.
JNJ- 74699157	Johnson & Johnson’s Janssen Pharmaceuticals and Wellspring Biosciences	Under early phase clinical trial for NSCLC and CRC.
Divarasib (GDC-6036)	Roche	Under early phase clinical trial for NSCLC and CRC.
BI 1823911	Boehringer Ingelheim	Under phase I clinical trial (NCT04973163) for NSCLC.
JDQ443	Novatis	Under phase III KontRASt-02 clinical trial for NSCLC.
LY3537982	Eli Lilly	Under phase I clinical trial for NSCLC and CRC.
**KARS G12D**		
MRTX1133	Mirati	Under early phase clinical trial for solid cancer.
HRS-4642	Shanghai Pulmonary Hospital	Under early phase clinical trial for solid cancer.
ASP-3082	Astellas	Under phase I clinical trial for solid cancer.
**KRAS G12V**		
BI 3706674	Boehringer Ingelheim	Under early phase clinical trial for solid cancer.
**Upstream signalling inhibitor-EGFR**		
Cetuximab	Eli Lilly	Approved by the FDA in 2004 for metastatic CRC and subsequently for head and neck squamous cell carcinoma with EGFR mutation.
Gefitinib	AstraZeneca	The first-line FDA-approved treatment with metastatic NSCLC whose tumours have EGFR exon 19 deletions or exon 21 substitution mutations.
TAGRISSO (AZD9291)	AstraZeneca	Treatment of patients with metastatic NSCLC that has progressed after EGFR tyrosine kinase inhibitor (TKI) therapy and is positive for the EGFR T790M mutation.
**Upstream signalling inhibitor-SOS**		
BAY-293	Bayer	A preclinical compound effectively disrupting the interaction between KRAS and its exchange factor SOS1, preventing KRAS switch from an "off" state to an "on" state.
BI-3406	Boehringer Ingelheim	A preclinical compound reducing GTP-KRAS formation.
**Upstream signalling inhibitor-SHP2**		
Jabi-3068	Jacobio Pharmaceuticals	The second SHP2 inhibitor targeting SHP2 in phase I/IIa clinal trial.
TNO155	Novartis	Under phase I clinical trials for RAS-mutant and RTK-dependent tumors.
RC-4630	Revolution Medicines/Sanofi	Under phase I for Advanced relapsed or refractory solid tumors.
RLY 1971	Relay Therapeutics	Under phase I for Advanced or metastatic solid tumors.
**Downstream signalling inhibitor-MEK1/2**		
Trametinib	GSK Pharmaceuticals	A PDA and EMA approved compound under phase II clinical trial for BRAF-mutant melanoma and NSCLC.
Binimetinib	Array Biopharma	A PDA and EMA approved compound under phase II clinical trial for BRAF-mutant melanoma and NSCLC.
Selumetinib	AstraZeneca	For the treatment of tumours associated with neurofibromatosis and various cancers.
Cobimetinib	Exelixis, Genentech	A PDA and EMA approved compound in combination with vemurafenib for unresectable or metastatic melanoma with a BRAFV600E or V600K mutation.
**Downstream signalling inhibitor-PI3K**		
Pictilisib	Genentech	Phase I study showed antitumor activity with acceptable safety, and a phase II study showed significant suppression of tumor cell proliferation
Copanlisib	Bayer	Under phase II for application for adult patients with relapsed follicular lymphoma who have received at least two prior systemic therapies.
Buparlisib	Novartis	The phase III trial BURAN compares buparlisib + paclitaxel to paclitaxel alone in patients with head and neck squamous cell carcinoma; The phase III trials BELLE-2 and BELLE-3 comparing buparlisib + fulvestrant with fulvestrant alone in patients with breast cancer both showed excessive side effects.
Apitolisib	Roche	Under phase II trials as a potential treatment for different solid tumours.
**Downstream signalling inhibitor-AKT**		
Lpatasertib	Genentech, Array BioPharma	Under phase III clinical trials for the treatment of metastatic castration-resistant prostate cancer and triple negative metastatic breast cancer.
Uprosertib	GSK Pharmaceuticals	Under phase I clinical trials to treat various cancers, including breast, cervical, endometrial, melanoma, and myeloma.

*Abbreviations*

*FDA* US Food and Drug Administration, *EMA* European Medicines Agency, *NSCLC* non-small cel lung cancer, *CRC* colorectal cancer

KRAS G12C inhibitors represent a significant breakthrough in the treatment of cancers driven by KRAS mutations. Sotorasib (AMG 510), also marketed as Lumakras, is the first FDA-approved inhibitor specifically target the KRAS G12C mutation, which is prevalent in NSCLC. This inhibitor covalently binds to the mutant cysteine residue (G12C) in KRAS, locking the protein in its inactive GDP-bound state and thereby repressing KRAS signaling [31, 163]. Clinical trials indicated that patients receiving Sotorasib treatment, who had previously undergone other anticancer therapies, experience increased progression-free survival and a favorable safety profile [164]. Adagrasib (MRTX1133), developed by Mirati Therapeutics, is another promising KRAS G12C inhibitor [165]. Clinical trials showed that combination therapy with Adagrasib and cetuximab results in longer median response duration and progression-free survival, along with fewer adverse response [166]. JNJ-74699157 and LY3499446 are additional KRAS G12C inhibitors that also bind specifically binding to the mutant cysteine at position 12 of the KRAS protein, locking it in its inactive GDP-bound state [167].

KRAS G12D is one of the most common KRAS mutations, particularly in PDAC (45%), CRC (45%), and NSCLC (17%) [162]. MRTX1133 is a promising inhibitor targeting KRAS G12D, demonstrating promising results in preclinical models [168]. HRS-4642 is an emerging drug candidate designed to inhibit the KRAS G12D mutation. A clinical trial in Shanghai Pulmonary Hospital showed HRS-4642 treatment led to objective responses in 2 of 9 KRAS G12D-driven NSCLC patients [169, 170].

Since KRAS activates the RAF/MEK/ERK pathway, MEK inhibitors have been explored as a therapeutic strategy. Drugs like Encorafenib, Trametinib and Cobimetinib inhibit MEK1/2, and are often used in combination with other therapies to improve efficacy [171, 172]. Another critical signaling pathway activated by KRAS mutations is the PI3K/AKT/mTOR pathway. Inhibitors targeting PI3K (e.g., Alpelisib), AKT (e.g., Ipatasertib), mTOR (e.g., Everolimus), are frequently used in combination with other drugs to target KRAS-driven cancers [173–175].

The intrinsic GTPase activity and GDP-GTP exchange rates can vary significantly among different RAS mutants, offering valuable insights for optimally target each specific mutant. Son of Sevenless 1 (SOS1) is a critical GEF for KRAS proteins, facilitating KRAS activation by promoting GDP-GTP exchange [176]. Inhibitors targeting the SOS1 such as BAY-293 and BI-3406, have been developed to block KRAS GDP-GTP switch, thus preventing the downstream pathway [177, 178].

These inhibitors target key points in KRAS pathway that are commonly dysregulated in cancers, providing significant opportunities for precision medicine. However, challenges such as drug resistance and toxicity highlight the need for ongoing research into combination therapies and biomarker-driven treatment strategies.

## Concluding remarks and perspectives

Tumor growth heavily depends on nutrient availability in the tumor microenvironment, with cancer cells undergoing metabolic reprogramming to sustain rapid proliferation, survive stress, and evade immune detection. In KRAS-driven cancers, metabolic reprogramming is a hallmark that supports rapid cell proliferation, survival, and tumor progression. Targeting cancer metabolism in KRAS-mutant tumors represents a promising therapeutic strategy, given their dependency on metabolic pathways like glycolysis, glutamine metabolism, and lipid biosynthesis. These multifaceted challenges highlight the need for innovative combination therapies that target multiple pathways, modulate the immune response, and address the dynamic resistance mechanisms of KRAS-driven tumors.

## Data Availability

No datasets were generated or analysed during the current study.
